# Trajectory Analysis of 6-DOF Industrial Robot Manipulators by Using Artificial Neural Networks

**DOI:** 10.3390/s24134416

**Published:** 2024-07-08

**Authors:** Mehmet Bahadır Çetinkaya, Kürşat Yildirim, Şahin Yildirim

**Affiliations:** 1 Faculty of Engineering, Department of Mechatronics Engineering, University of Erciyes, Kayseri 38039, Turkey; cetinkaya@erciyes.edu.tr; 2 Graduate School of Natural and Applied Sciences, University of Erciyes, Kayseri 38039, Turkey; kursatyildirim17@icloud.com

**Keywords:** industrial robot manipulator, trajectory planning and analysis, artificial neural networks, learning algorithms

## Abstract

Robot manipulators are robotic systems that are frequently used in automation systems and able to provide increased speed, precision, and efficiency in the industrial applications. Due to their nonlinear and complex nature, it is crucial to optimize the robot manipulator systems in terms of trajectory control. In this study, positioning analyses based on artificial neural networks (ANNs) were performed for robot manipulator systems used in the textile industry, and the optimal ANN model for the high-accuracy positioning was improved. The inverse kinematic analyses of a 6-degree-of-freedom (DOF) industrial denim robot manipulator were carried out via four different learning algorithms, delta-bar-delta (DBD), online back propagation (OBP), quick back propagation (QBP), and random back propagation (RBP), for the proposed neural network predictor. From the results obtained, it was observed that the QBP-based 3-10-6 type ANN structure produced the optimal results in terms of estimation and modeling of trajectory control. In addition, the 3-5-6 type ANN structure was also improved, and its root mean square error (RMSE) and statistical R^2^ performances were compared with that of the 3-10-6 ANN structure. Consequently, it can be concluded that the proposed neural predictors can successfully be employed in real-time industrial applications for robot manipulator trajectory analysis.

## 1. Introduction

Industrial robots are capable of performing many different processes and operations accurately and do not need complex supporting elements such as humans need. Robot manipulators are powerful electromechanical systems that provide effective solutions to the recent industrial applications such as picking, placing, packing, painting, and welding. Especially, robot manipulators with multi-DOF are widely used at all stages of production processes such as automation systems due to their ability to perform industrial operations effectively without the need of humans. In addition, significant advantages in terms of occupational health and safety have been provided by switching from the operator-assisted production model to robot manipulator-supported structures in processes involving chemicals and carcinogens.

One of the most important problems encountered in robot manipulator systems is the ability to achieve high-accuracy positioning for even large disturbances such as mechanical friction of the mechanical parts, ambient ventilation, and mechanical strength misalignment. Due to the basic disturbances faced in the production processes, it is crucial to use artificial intelligence-based predictors for trajectory analysis as an inverse kinematic solution. In this study, ANN-based detailed trajectory analyses were carried out to enable the robot manipulator end processor to monitor the full trajectory with the highest accuracy during the denim fabric grinding process. To our best knowledge, for the relevant research area, DBD, OBP, QBP, and RBP-based nonlinear artificial neural network structures were first improved in the literature within the scope of this study.

In order to perform the position control of a robot manipulator, inverse kinematic equations or forward kinematic equations can be used. However, forward kinematic equations require longer calculation times and also complex mathematical analysis such as the Gaussian elimination approach. Due to these important disadvantages of forward kinematic equation-based analysis, inverse kinematic equation-based analysis is frequently preferred. Namely, the equations that were found for solving the inverse kinematic of the 6-DOF robot manipulator can directly be used to drive the robot to a desired position.

In the literature, there are several studies including different application areas of robot manipulator systems. Saad et al. developed an ANN-based adaptive controller for robot manipulator applications in [1]. A geometric algorithm for fixed directional working area calculation in 6-PRRS structured robot manipulators was proposed by Boney and Ryu, and its applications were analyzed [2]. In a study conducted by Bonev et al. [3] on the kinematics of parallel robot manipulators with linear sensors and optimal positioning, a closed-form solution approach was developed. Detailed research on the singularities of 3-DOF parallel robot manipulator mechanisms was carried out by Boney and his research group [4]. Tombul and Sarıtaş presented detailed research that was conducted on inverse kinematic calculations and trajectory planning for a five-axis Edubot robot [5]. On the other hand, Khayati et al. carried out analyses on multi-stage position/force control for friction-constrained robotic systems [6]. Lessard et al. carried out analyses on static balancing optimization by designing position and force controllers for biomedical applications, especially three-dimensional ultrasound imaging [7]. Karahan obtained the robot inertia parameters by performing detailed dynamic model analysis for the Staubli RX-60 robot in his master’s thesis [8]. In another study, Janvier et al. performed detailed performance analyses for medical robotic three-dimensional ultrasound imaging systems [9]. An updated formulation and its related applications improved for a KUKA-type industrial robot manipulator were presented by Bigras et al. [10]. Moreover, Yu et al. proposed a geometric approach for accuracy analysis of 3-DOF planar parallel robots [11]. Furthermore, accuracy analysis of a 3-DOF planar parallel robot structure was performed by Briot and Bonev [12]. On the other hand, parallel kinematic analyses were carried out by Liu et al. on a two-joint robot structure, and then detailed analyses on adaptability, error optimization, and size optimization were realized [13]. Furthermore, accuracy analysis of a 3-DOF planar parallel robot structure was performed by Briot and Bonev [14]. A novel controller structure combining the adaptive neural networks and proportional-integral-derivative (PID) control approach was presented by Perez et al. to optimize the trajectory behavior of robot manipulators [15]. An ANN-based method that was able to adapt to the characteristics of both known and unknown trajectories of 6-DOF robot manipulators was improved by Tang et al. [16]. Pham and Wang improved a robust radial basis function neural network (RBFANN)-based adaptive control approach to optimize the joint position control and trajectory tracking control of robot manipulators [17]. An inverse dynamic model estimation approach based on ANNs was proposed by Moldovan et al. in study [18] to optimize the trajectory performance of a 6-DOF robot manipulator. Mahajan et al. improved an ANN-based structure to optimize the trajectory tracking of a 2-DOF robotic arm by using the inverse kinematics equations [19]. Moreover, Son et al. proposed an adaptive ANN model that was strengthened by differential evolution algorithm in order to optimize the non-linear dynamics of a 5-DOF robot manipulator [20]. An ANN-based control process with an optimal number of hidden nodes and less computation was proposed by Liu et al. to overcome the system uncertainties and track the trajectory of the robot manipulator with high accuracy [21]. Şeker et al. introduced a convolutional neural network (CNN)-based long short-term memory (LSTM) model to obtain a high-precision performance especially in the prediction of lever-up actions and tested its performance on a real UR10 robot [22]. A radial basis ANN structure including a fuzzy sliding mode was improved by Wang et al. in [23] to obtain an optimized trajectory control. Truong et al. proposed a novel adaptive tracking ANN with a deadzone robust compensator for industrial robot manipulators to achieve the high-precision position tracking performance [24]. In addition, a Lyapunov function-based control scheme and a RBFANN structure was proposed by Yang et al. to optimize the trajectory tracking process of a robot manipulator [25]. Nubert et al. presented a robust approach that combined the model predictive control algorithm and neural networks to provide safe and fast tracking on robot manipulators [26]. On the other hand, Elsisi et al. proposed an ANN-based modified adaptive tuning algorithm in order to track the trajectories of robot manipulator arms with high accuracy [27]. Song et al. constructed a 6-DOF robot experimental platform and then optimized its entire trajectory planning framework via a radial basis function neural network [28]. In another study, Liu et al. proposed a neural network-based mechanical adaptive control method to optimize the motion characteristics of a robot manipulator [29]. A novel approach about the kinematic and singularity analyses was proposed by Shi et al. for a 7-DOF redundant manipulator with three consecutive parallel axes [30]. A multi-objective design mechanism was introduced by Kouritem et al. to determine the optimal material type and optimal physical dimensions of the robot arm to withstand loads at vulnerable locations using stress analysis [31]. A hybrid approach consisting of back propagation neural network and genetic algorithms was improved in [32] by Qie et al. in order to plan and optimize the trajectory of a redundant robot manipulator. Lu et al. proposed a novel approach consisting of inverse kinematics and neural networks to provide inverse kinematic solutions with high accuracy [33]. Bao et al. improved an adaptive trajectory tracking control scheme that included both a radial basis function neural network structure and a computed torque control (CTC) method to optimize robot manipulator systems [34]. In work [35], Shi et al. introduced a learning control framework that was able to provide an optimal dynamic tracking behavior for robot manipulators. Finally, Xu et al. improved a novel hybrid neural network-based learning control method to obtain an accurate approach for trajectory tracking of complex robot manipulators [36].

This article presents the following contributions to the literature.

- The nonlinear artificial neural network structures of DBD, OBP, QBP, and RBP were improved and then presented to the literature as effective inverse kinematic analysis approaches.
- The simulation results obtained for both 3-10-6 and 3-5-6 type ANN structures provided a detailed database for multi-DOF robot manipulator manufacturers and researchers, especially in the textile sector.

## 2. Materials and Methods

In this study, detailed trajectory planning and analysis of a real-time 6-DOF industrial robot manipulator system were realized by using DBD, OBP, QBP, and RBP-based improved ANN network structures. The properties of the analyzed industrial robot manipulator system and the structure of the improved ANN networks can be explained in detail as follows.

### 2.1. Industrial Robot Manipulators

**Figure 1:**
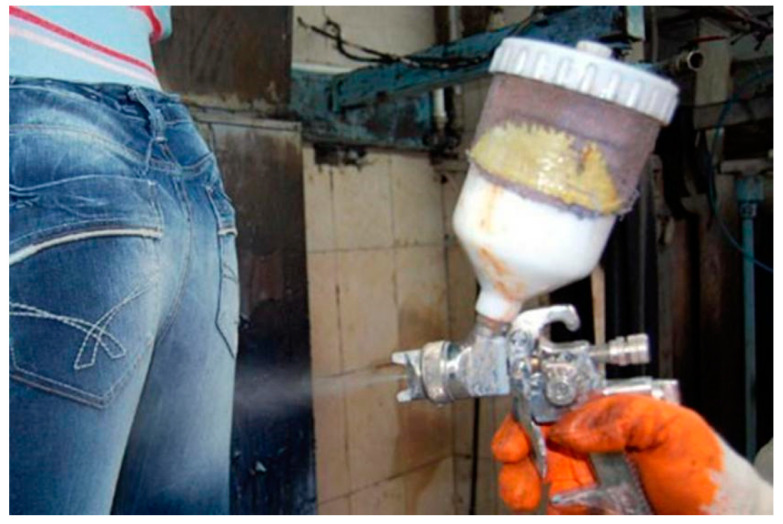
Classical human-based chemical spraying process on denim textile.

One of the most important stages in the production of jeans trousers is the chemical spraying process. In the classical human-based chemical spraying process, which is shown in Figure 1, undesirable effects such as health problems and color tone differences may occur.

**Figure 2:**
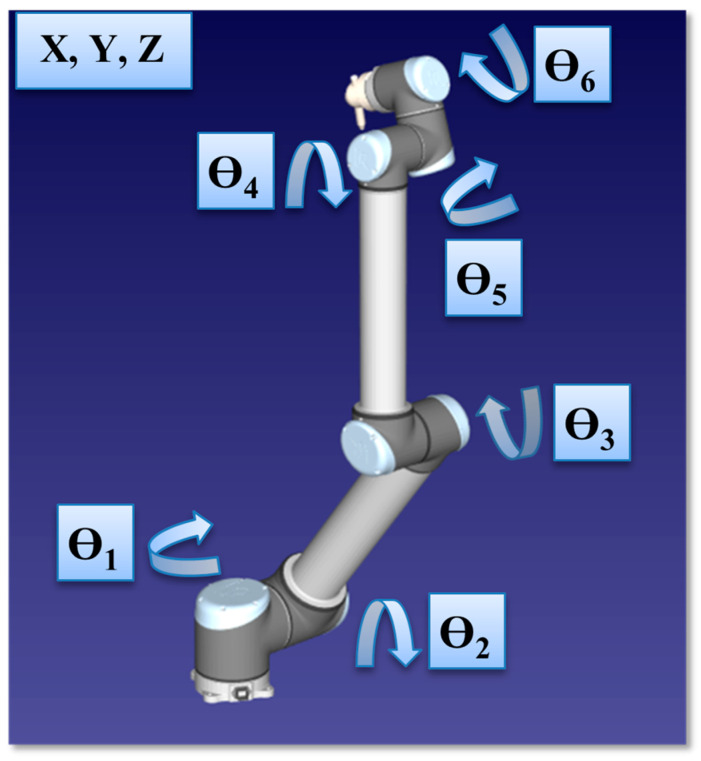
Representation of the proposed 6-DOF industrial robot and its rotation axes.

In order to overcome the undesirable effects of the human-based spraying process, industrial robot manipulators can be preferred. In this study, first, kinematic analyses of a Universal Robots UR5e model 6-DOF robot manipulator for chemical spraying were performed according to [37], and then ANN-based trajectory optimization was carried out. The structure and rotation axes of the 6-DOF industrial robot analyzed in this study are shown in Figure 2. As seen from the figure, the 6-DOF industrial robot structure analyzed in this study had six joint angles of $\theta_{1},\theta_{2},\theta_{3},\theta_{4},\theta_{5}$, and $\theta_{6}$. The robot manipulator analyzed in this study also consisted of three types of rotations including pitch, yaw, and roll angles, which corresponded to the orientations of the end effector. In order to construct the orientation process of the end effector, a coordinate system was attached to the body of the manipulator via RoboAnalyzer v8.0.1(R) software, and then orientation was optimized by using the same software. After the orientation process was constructed, the accuracy and robustness of the proposed approach were tested in terms of inverse kinematic.

The initial position of the robot manipulator’s end effector was determined as a local area on the denim jean. The initial position information was defined via RoboAnalyzer v8.0.1(R) software as including the kinematics of the 6-DOF robot manipulator. These randomly produced position data were used as input variations of the proposed ANN predictors. In addition, the final position information was also determined depending on the initial positions.

The technical properties and parameters of the industrial robot manipulator analyzed and designed within the scope of this study are shown in Table 1 [38]. As understood from the table, the 6-DOF industrial robot structure analyzed in this study was capable of contributing significantly to production speed and precision. In addition, the fact that the robot manipulator had (±360°) rotation angle in all its joints meant that the points it could reach in the working space were at the maximum level. In addition, since the operating noise level was too low, even if many robots were used in a production line, noise-related disturbances would not occur, and the total system performance would not negatively be affected.

The Denavit–Hartenberg (D-H) parameters representing the kinematic parameters of the robot manipulator are shown in Table 2 below [38]. Each joint of the robot manipulator had the feature of a rotary joint, and structurally, there was no joint misalignment or second adjacent axis angle. Therefore, it was obvious that this structure would provide an important advantage, especially in exact positioning. 

**Figure 3:**
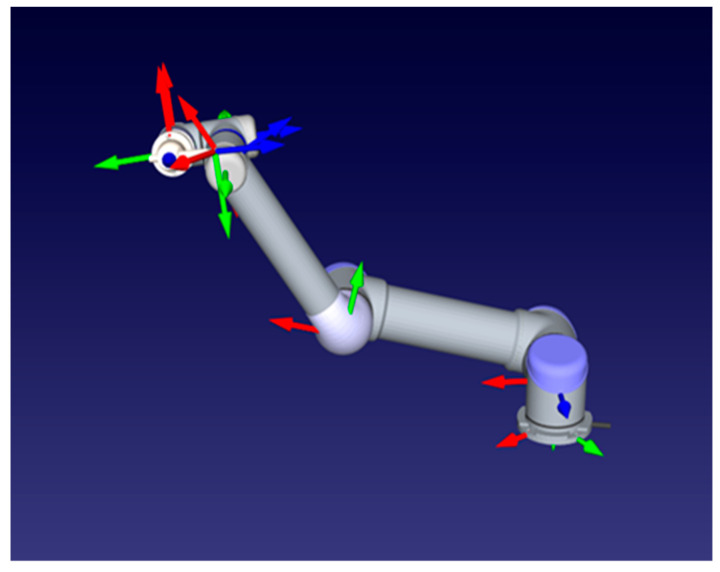
Robot manipulator and each joint axes sets.

The industrial robot manipulator structure designed in this study consisted of in total six joints, including the body, shoulder, elbow, and wrist joints, as seen in Figure 3.

### 2.2. Artificial Neural Networks

**Figure 4:**
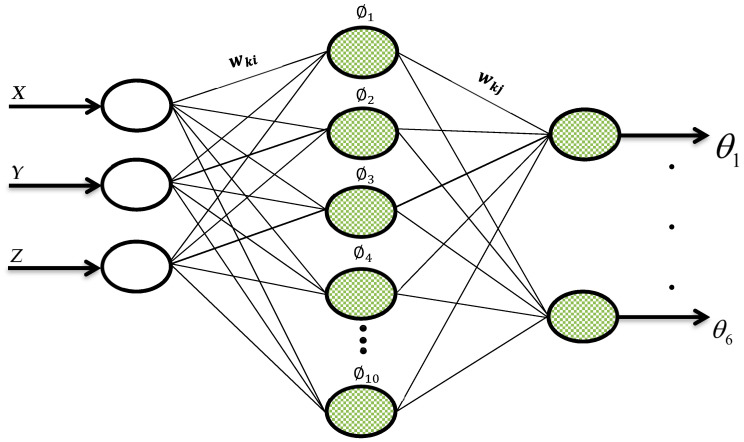
The proposed artificial neural network representation.

ANNs are nonlinear mapping systems with a structure based on the principles observed in biological nervous systems. A basic ANN structure consists of an input layer ($x_{i}$), weights ($w_{ki},w_{kj}$), an activation level ($\phi_{j}$), and an output layer ($y_{j}$) as represented in Figure 4. 

In this network structure, the input vector is updated via a Gaussian activation function that performs a nonlinear transformation and is defined in Equation (1).

(1) $$\phi_{j}=\exp\left[-{\left(\frac{\left\|x_{i}-g_{c}\right\|}{\sigma }\right)}^{2}\right]$$

where $x_{i}$ is the input vector; $g_{c}$ is the center of the jth Gaussian function; σ>0 is the spread constant parameter; and finally, $\left\|x_{i}-g_{c}\right\|$ defines the Euclidean distance between the two relevent vectors. Afterward, in the second level, each output of the network can be calculated via a linear transformation as defined in Equation (2).

(2) $$y_{j}=\sum_{j=1}^{N}w_{kj}.\;\phi_{j}$$

where N is the number of hidden layers and $w_{kj}$ are the weight values between the hidden and output neurons.

In this study, analyses were carried out by using an ANN network structure including two hidden layers. For rotation angle analysis of each joint of the robotic manipulator system, ANN network structures including 3 input layer cells, in total 10 hidden layer cells, and 6 output layer cells were improved (the 3-10-6 ANN network structure). The spread constant parameter directly affected the approximating performance of the ANN network structure. In order to find the most appropriate value, the simulations based on test data (30% of the experimental data) were performed for 0.1 step sizes in the range (0, 1] in line with the problem structure, and the optimal value of σ that produced the minimum root mean squared error (RMSE) was obtained as 0.1. 

In order to determine the optimum ANN network structure, the performances of the delta-bar-delta (DBD), online back propagation (OBP), quick back propagation (QBP), and random back propagation (RBP) were analysed.

DBD learning algorithm is an adaptive method in which each weight has its own learning rate. The learning rates are updated based on the change in the sign of the gradient on consecutive iterations. If the sign of the gradient does not change, the step size will be increased linearly. In contrast, if the gradient sign changes, the learning rate will be decreased exponentially. In some cases, DBD seems to learn much faster than non-adaptive methods. In the DBD learning algorithm, the learning rates are updated by using Equation (3):

(3) $$\Delta w_{ij}(t+1)=\left\{ \begin{array}{ll} K, & if\;\;\delta^{\prime }(t-1)\;\delta (t)>0\\ -\phi \eta (t), & if\;\;\delta^{\prime }(t-1)\;\delta (t)<0\\ 0, & else \end{array}\right.$$

where K and ϕare the positive constants; η is the learning rate; and finally, $\delta (t)=\frac{\partial E(t)}{\partial w_{ji}(t)}$, in which E(t) represents the instantaneous sum of the squared errors. In the DBD learning algorithm, the value of the η parameter was taken as 0.1.

BP is the most widely used training algorithm for ANNs. The weights of the network were set as represented in Equation (4).

(4) $$\Delta w_{ij}(t)=-\eta \frac{\partial E(t)}{\partial w_{ij}(t)\;}\;\;+\;\;\alpha \;\Delta w_{ij}(t-1)$$

where η is the learning rate, E(t) represents the instantaneous sum of the squared errors, and α determines the momentum term. In the simulations, the values of the η and α parameters were taken as 0.1 and 0, respectively. On the other hand, in the OBP learning algorithm, unlike the BP algorithm, the weight values were updated after the model was presented to the ANN. The OBP algorithm with a randomly selected input layout order makes the learning process stochastic and is preferred in most cases.

QBP is an improved version of BP that uses the hyperbolic tangent function instead of the sigmoid function. In other words, in QBP, all the hidden layer neurons and all the output layer neurons use the hyperbolic tangent function while training the network. The weights of the network are updated by using Equation (5) at each iteration:

(5) $$\Delta w(t)=\frac{s(t)}{s(t-1)-s(t)\;\;}\;\;\;\Delta w(t-1)-\eta s(t)$$

where $s(t)=\frac{\partial E(t)}{\partial w(t)\;}$, in which E(t) represents the instantaneous sum of the squared errors and η represents the learning rate, which was defined as 0.1. 

RBP is an adaptive learning rate method where weight updates are based on the sign of the local gradients, not their magnitudes. In RBP, each weight has its own step size or update value, which varies with time according to Equation (6):

(6) $$\Delta w_{ij}(t+1)=\left\{ \begin{array}{ll} \eta^{+}\Delta w_{ij}(t-1), & if\;\;\frac{\;\partial E(t-1)}{w_{ij}(t-1)}\frac{\;\partial E(t)}{w_{ij}(t)}>0\;\\ \eta^{-}\Delta w_{ij}(t-1), & if\;\;\frac{\;\partial E(t-1)}{w_{ij}(t-1)}\frac{\;\partial E(t)}{w_{ij}(t)}<0\\ \Delta w_{ij}(t-1), & else \end{array}\right.$$

where η defines the learning rate with the rule of $0<\eta^{-}<1<\eta^{+}$ and E(t) can be defined as the instantaneous sum of the squared errors. 

**Figure 5:**
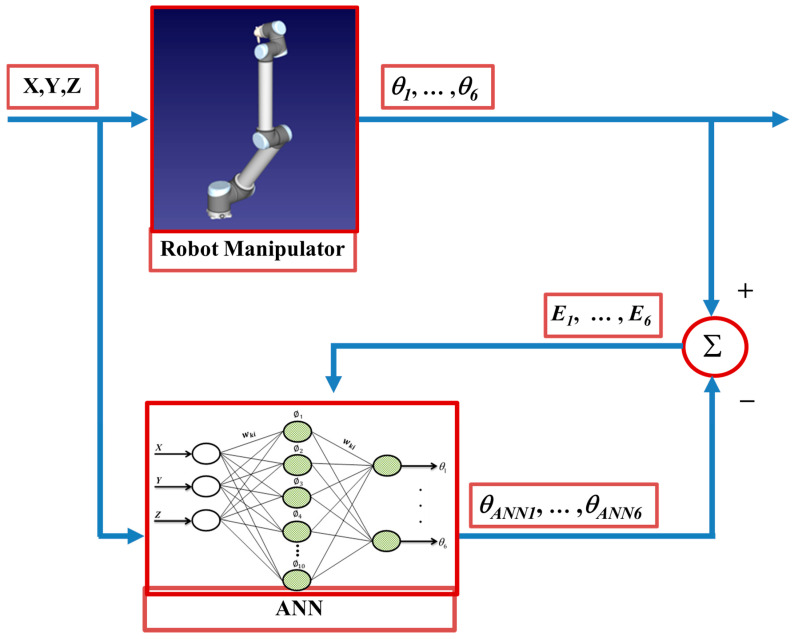
ANN-based modeling of a robot manipulator.

The structure of the ANN-based analysis carried out to determine and optimize the six joint angles of a specifically determined trajectory for a robot manipulator is given as a block diagram in Figure 5. As seen from the figure, the learning process was optimized by using the RMSE values that were the difference between the actual angular values and the angular values produced by the ANN. The expression of the RMSE can be defined as given in Equation (7):

(7) $$RMSE=\sqrt{\frac{1}{N}\sum_{i=1}^{MI}{\left(Y_{Actual}-Y_{ANN}\right)}^{2}}$$

where *MI* defines the maximum number of iterations, and *N* is the number of data that is equal to maximum number of the test data set.

## 3. Simulation Results

In the simulations carried out, the ANN structure consisting of 3 input cells (position of the end function—X, Y, Z), 10 hidden layer cells, and 6 output layer cells (joint angles—$\theta_{1},\theta_{2},\theta_{3},\theta_{4},\theta_{5}$, and $\theta_{6}$) was formed. Furthermore, randomly selected 70% of the entire data set was used for the training of the ANN, and the remaining 30% of the data were used for test phase. In all the simulations the iteration number was taken as 1,000,000. 

**Figure 6:**
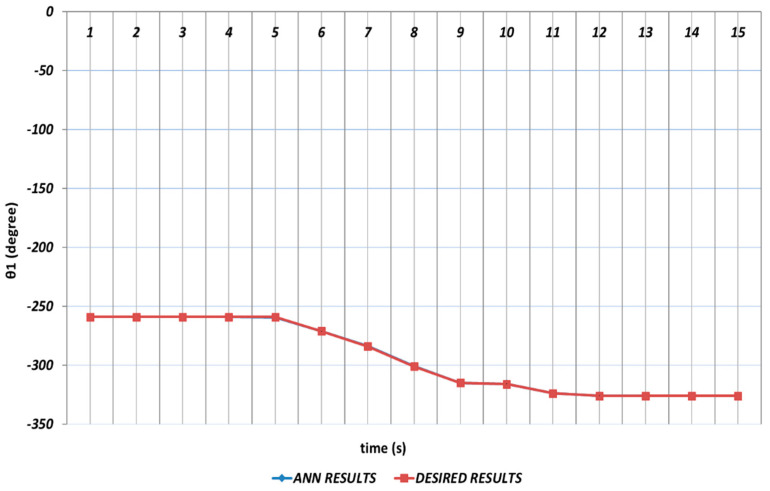
DBD learning algorithm-based prediction results for the first joint angle.

**Figure 7:**
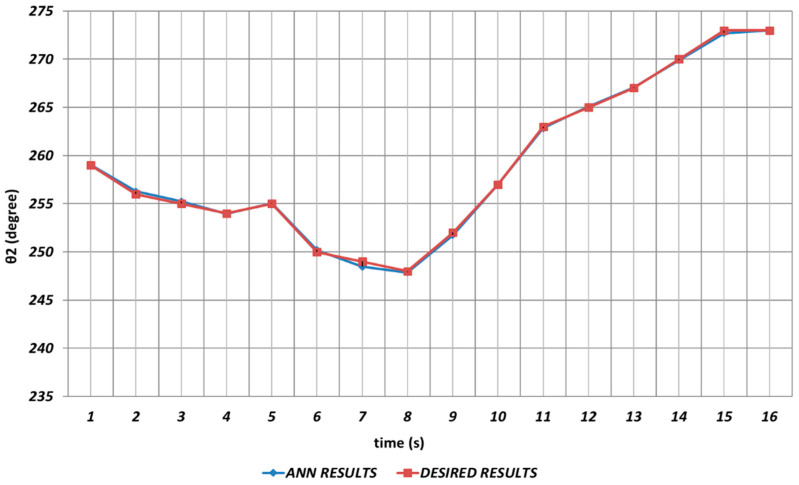
DBD learning algorithm-based prediction results for the second joint angle.

**Figure 8:**
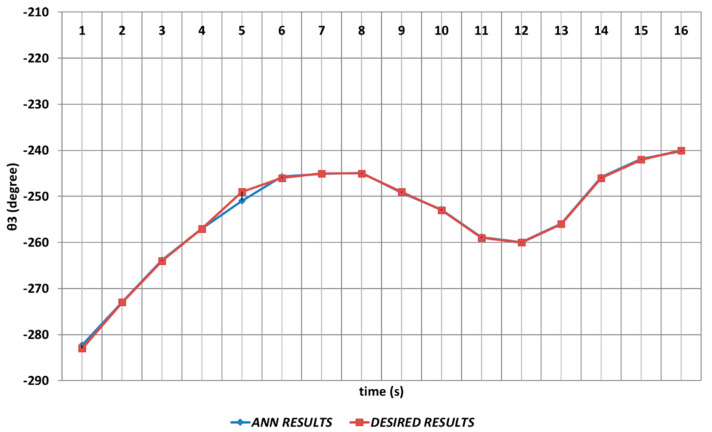
DBD learning algorithm-based prediction results for the third joint angle.

**Figure 9:**
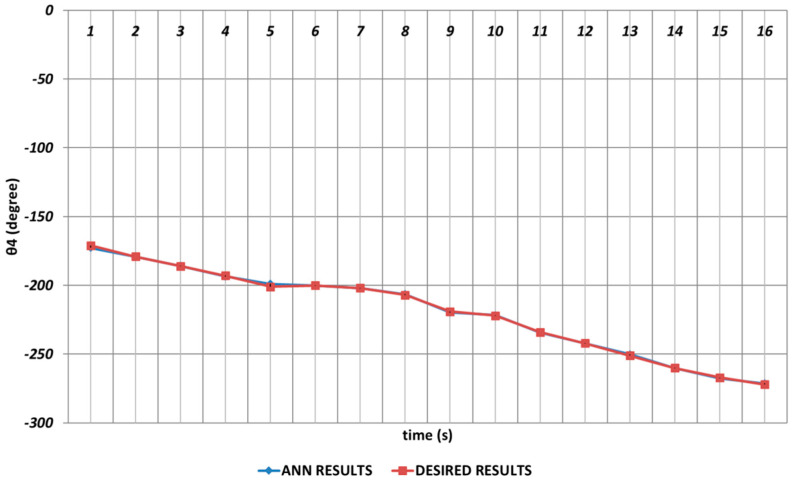
DBD learning algorithm-based prediction results for the fourth joint angle.

**Figure 10:**
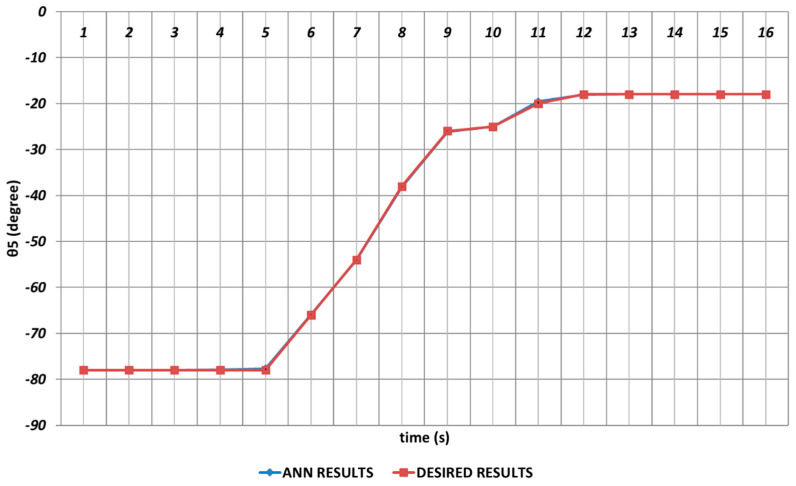
DBD learning algorithm-based prediction results for the fifth joint angle.

**Figure 11:**
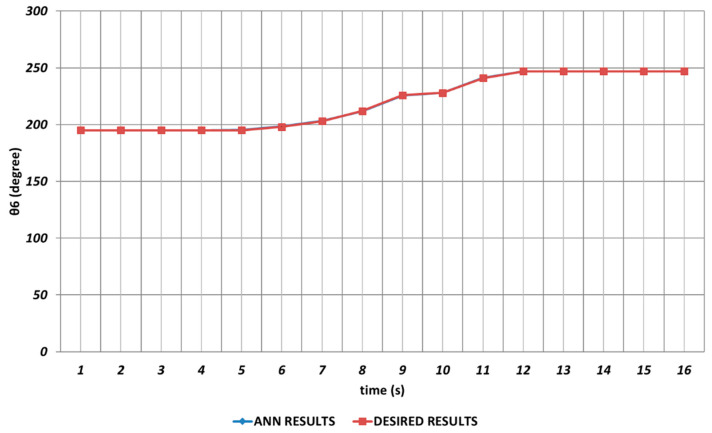
DBD learning algorithm-based prediction results for the sixth joint angle.

In Figure 6, Figure 7, Figure 8, Figure 9, Figure 10 and Figure 11, DBD learning algorithm-based simulations results are presented. As seen from Figure 6, which represents the modeling performance for $\theta_{1}$, the DBD algorithm was able to predict the theoretical data with high accuracy. Although there were instantaneous angular changes at the fifth and ninth seconds, the DBD could follow these changes successfully.

Figure 7 shows the DBD-based prediction results for $\theta_{2}$. Since it was used as the shoulder angle, it was seen that there were significant prediction errors in the intervals of the 2nd and 4th seconds, the 6th and 8th seconds, and similarly the 14th and 16th seconds. Namely, DBD-based estimation seemed a bit insufficient in predicting instantaneous changes in the time intervals mentioned. This situation showed that an unexpected position difference would occur in serial chemical application between the fabric where the chemical would be sprayed and the ideal position. 

The DBD-based prediction results of $\theta_{3}$, which performed the elbow function in the human structure, are demonstrated in Figure 8. Since the shoulder angle appearing after the angular change was used as the next period angle at the same time, instantaneous changes in the time intervals of the first and second seconds and also the fourth and sixth seconds were observed. It could be concluded that significant spray errors would be observed, especially in the time interval of the fourth and sixth seconds, in serial chemical applications. On the other hand, despite the errors that occurred during instantaneous angular changes, the prediction performance seemed successful outside of these two time intervals.

The $\theta_{4}$ joint angle corresponded to the rotation angle of the robot gripper. When the DBD-based prediction results were analyzed, it could be emphasized that the theoretical data could be predicted with high accuracy even in the intervals of the 4th and 6th seconds and the 8th and 10th seconds at which instantaneous angular changes appeared.

Especially the fifth joint angle had an important role in positioning structure and rotation angle, and even small prediction errors for $\theta_{5}$ were not acceptable. First, instantaneous angular changes occurred in the intervals of the 4th and 5th seconds and similarly in the 10th and 12th seconds, as seen in Figure 10. As a result of these instantaneous angular changes, it was seen that deviations with too small magnitudes were occurring in the DBD-based prediction results. When the prediction performance was evaluated in a general manner, it could be expressed that DBD could successfully predict the theoretical data for $\theta_{5}$.

Figure 11 shows the prediction results obtained for $\theta_{6}$, which was the last rotation angle of the robot manipulator. It was seen that small prediction errors were almost non-existent for the sixth joint angle. The reason of this could be expressed as no instantaneous angular changes were occurring during the entire time interval.

**Figure 12:**
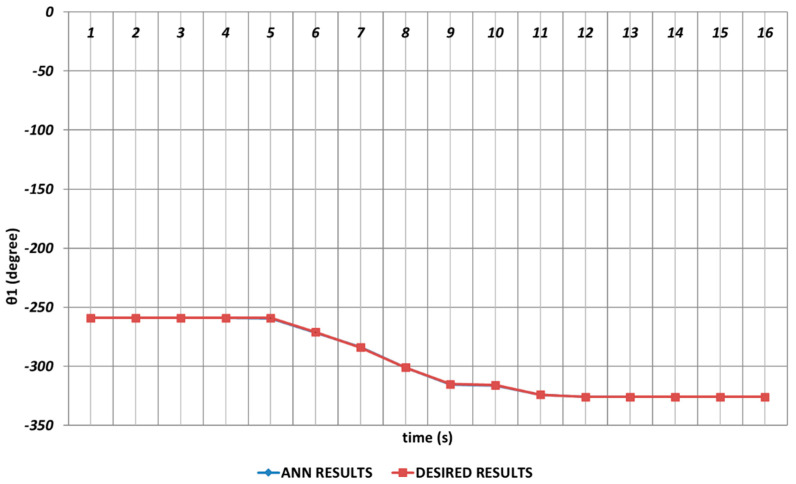
OBP learning algorithm-based prediction results for the first joint angle.

**Figure 13:**
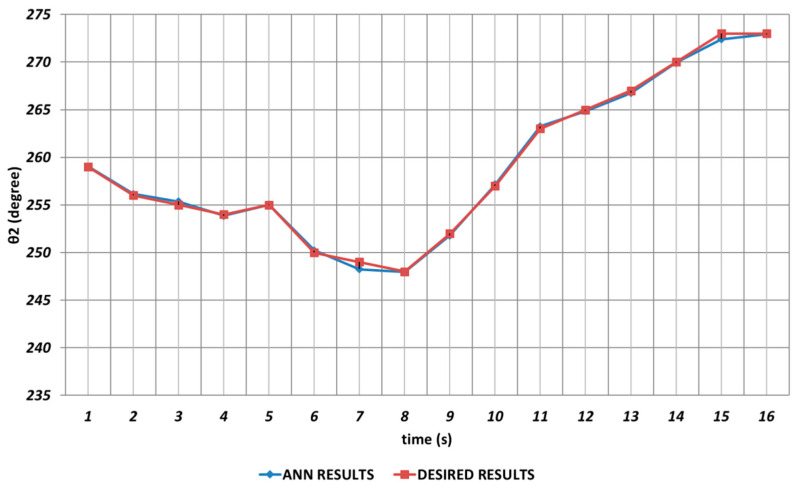
OBP learning algorithm-based prediction results for the second joint angle.

**Figure 14:**
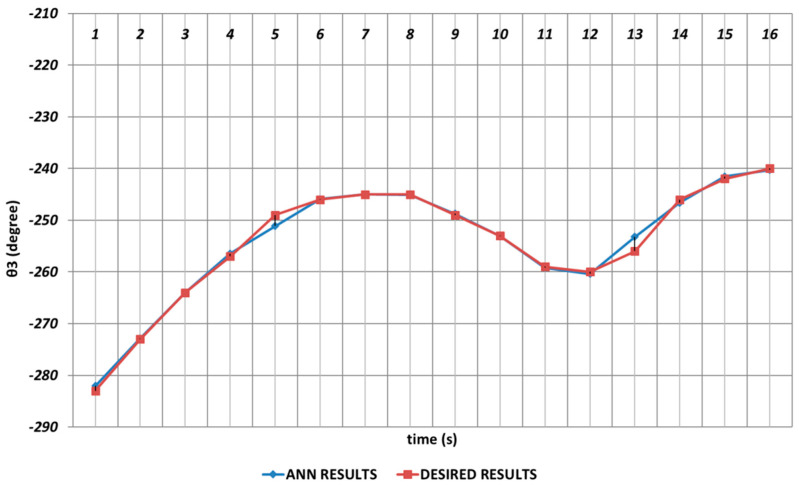
OBP learning algorithm-based prediction results for the third joint angle.

**Figure 15:**
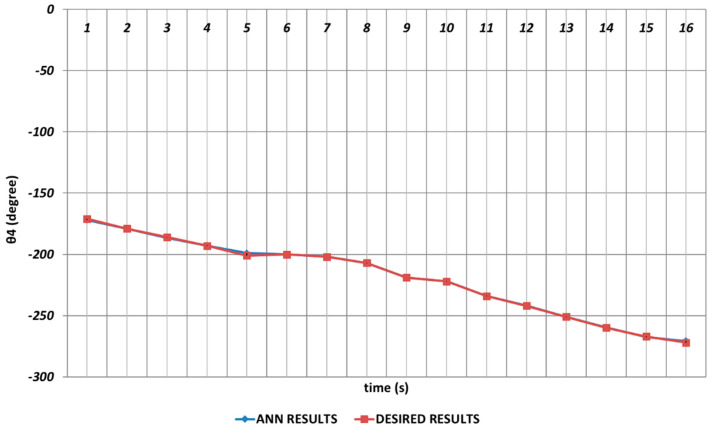
OBP learning algorithm-based prediction results for the fourth joint angle.

**Figure 16:**
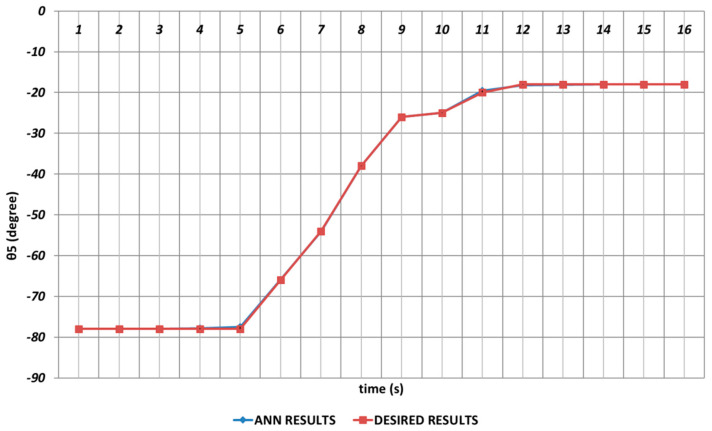
OBP learning algorithm-based prediction results for the fifth joint angle.

**Figure 17:**
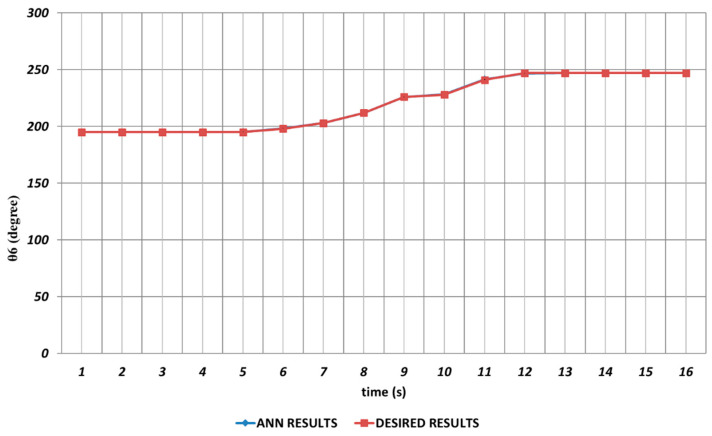
OBP learning algorithm-based prediction results for the sixth joint angle.

The analysis results obtained for the OBP learning algorithm are given in Figure 12, Figure 13, Figure 14, Figure 15, Figure 16 and Figure 17. The prediction performance for the $\theta_{1}$ joint angle is presented in Figure 12. From the figure, it is seen that OBP was able to produce superior prediction results for the first joint angle, which was the body rotation angular of the robot manipulator. In other words, no instantaneous angular changes were occurring, and the OBP-based results converged to the theoretical results with high accuracy during the entire time interval.

In Figure 13, the prediction performance of the OBP learning algorithm is shown for the $\theta_{2}$ joint angle, which corresponded to the shoulder angle. It was seen that there were significant prediction errors in the time intervals of the 2nd and 4th seconds, the 6th and 8th seconds, and the 11th and 16th seconds due to the instantaneous angular changes. These angular differences would cause the chemical spraying process to be carried out with incorrect angle values.

Figure 14 demonstrates the OBP-based prediction results obtained for $\theta_{3}$, which was the third joint angle of the robot manipulator. Since the shoulder angle obtained after the angular change was used as the next period angle at the same time, it was observed that significant deviations from the theoretical data appeared in the time intervals of the 1st and 2nd seconds, the 4th and 6th seconds, and also the 11th and 16th seconds. As a result, it could be emphasized that the prediction performance for the third joint angle seemed insufficient when the OBP learning algorithm was used. In contrast, OBP was able to provide better prediction performances outside of the time intervals that included instantaneous angular changes.

In Figure 15, the simulation results produced by OBP in the prediction of the $\theta_{4}$ joint angle are presented. From the results, it could be expressed that the rotation angle of the robot gripper could successfully be predicted with high accuracy via the OBP learning algorithm. However, although instantaneous angular changes had no significant effect, it was seen that prediction errors were occurring between the theoretical and ANN approach at certain rates in the time intervals of the 4th and 6th seconds and the 14th and 16th seconds.

The $\theta_{5}$ joint angle was extremely important for the positioning of the end effector used in the chemical spray system. Due to its critical role in optimizing the positioning structure of the entire system, even small prediction errors were not acceptable for this angle as mentioned before. As seen from the prediction results given in Figure 16, deviations with too small magnitudes occurred especially in the time intervals of the 3rd and 6th seconds and the 10th and 13th seconds.

As seen from Figure 17, which represents the OBP-based prediction results obtained for $\theta_{6}$, an excellent prediction performance could be provided. The limited number of instantaneous position changes in the theoretical data ensured an effective prediction process during the entire time interval.

**Figure 18:**
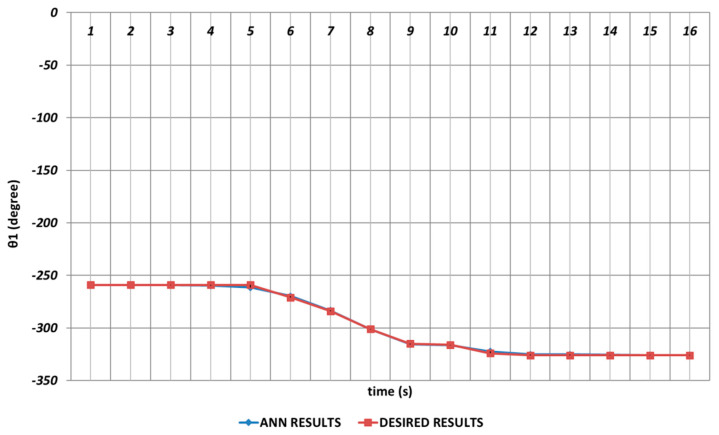
QBP learning algorithm-based prediction results for the first joint angle.

**Figure 19:**
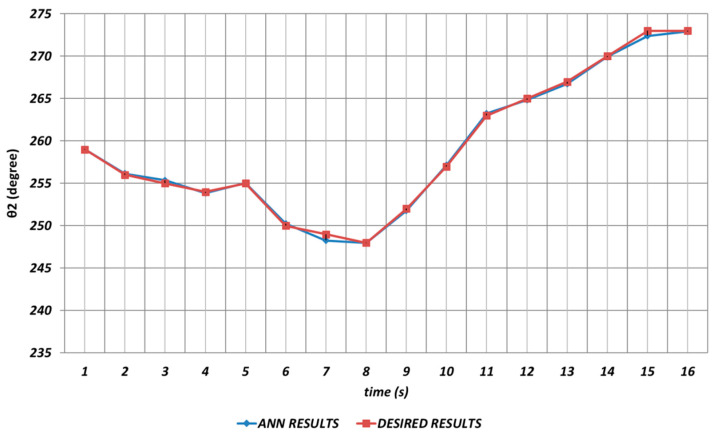
QBP learning algorithm-based prediction results for the second joint angle.

**Figure 20:**
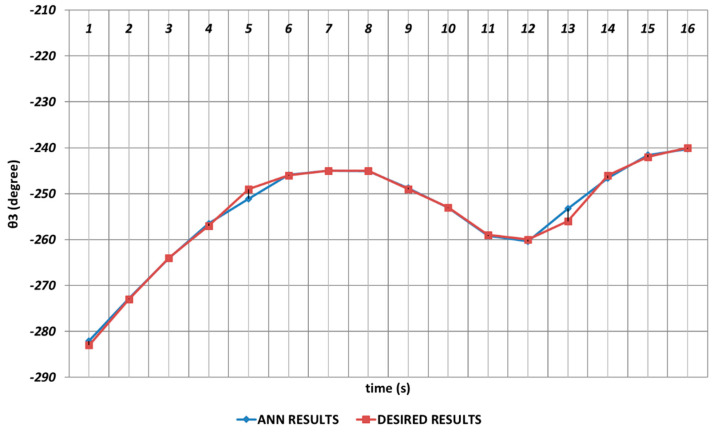
QBP learning algorithm-based prediction results for the third joint angle.

**Figure 21:**
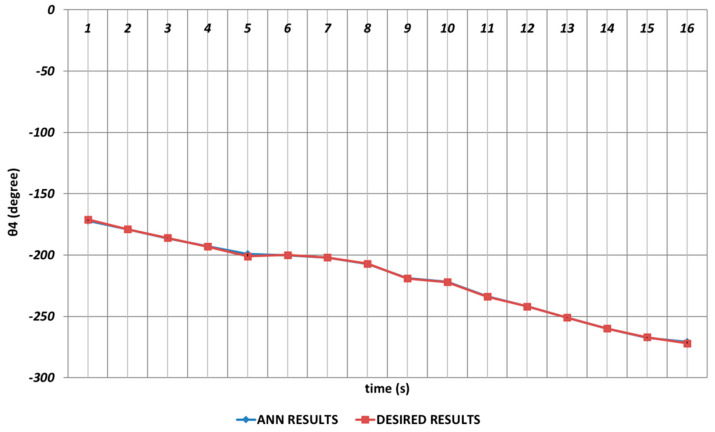
QBP learning algorithm-based prediction results for the fourth joint angle.

**Figure 22:**
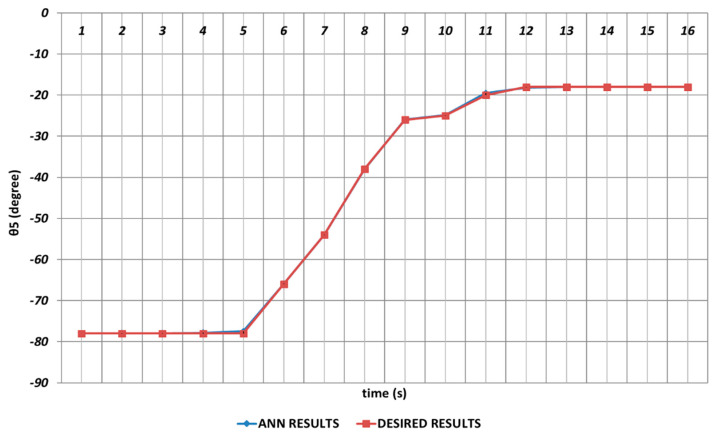
QBP learning algorithm-based prediction results for the fifth joint angle.

**Figure 23:**
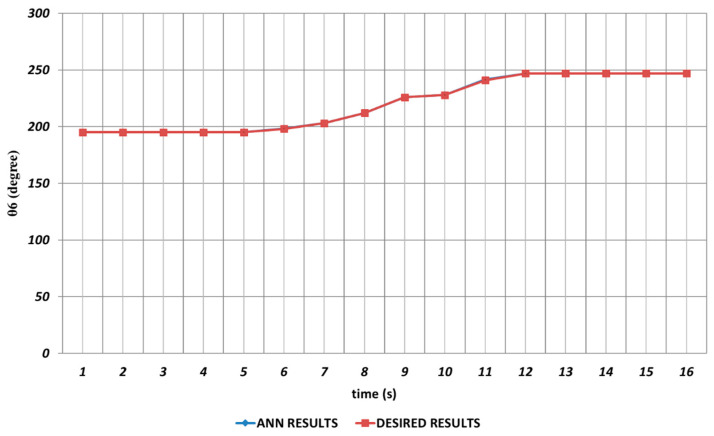
QBP learning algorithm-based prediction results for the sixth joint angle.

The prediction performance of the QBP learning algorithm in terms of the joint angles is represented in Figure 18, Figure 19, Figure 20, Figure 21, Figure 22 and Figure 23. Figure 18 proves that QBP was able to provide an excellent prediction performance for $\theta_{1}$ during the entire time interval, including instantaneous angular changes.

QBP-based prediction results for the robot manipulator shoulder angle $\theta_{2}$ are shown in Figure 19. As can be seen from the figure, there were significant prediction errors especially in the time intervals of the 2nd and 4th seconds, the 6th and 8th seconds, and the 14th and 16th seconds due to the effects of instantaneous angular changes. In other words, the prediction results for $\theta_{2}$ should not be used directly in serial chemical applications.

Figure 20 demonstrates the QBP-based prediction results for the $\theta_{3}$ joint angle, which simulated the elbow function of humans. Due to the instantaneous angular changes in the time intervals of the 1st and 2nd seconds, the 4th and 6th seconds, and the 11th and 14th seconds, the theoretical data could not be predicted successfully. As a result, it could be emphasized that the prediction for the third joint angle was not very effective, and the performance of the QBP seemed insufficient at the relevant time intervals. 

In Figure 21, the QBP prediction results obtained for the $\theta_{4}$ joint angle are given. In general, it could be said that the QBP could successfully converge to the theoretical data except the time intervals of the 4th and 6th seconds and the 14th and 16th seconds. On the other hand, it could also be stated that the deviations from the theoretical data were occurring with too small magnitudes in the relevant time intervals. 

Due to its importance as mentioned before, the prediction of the $\theta_{5}$ joint angle with high accuracy was crucial. Figure 22 represents the prediction performance of the QBP learning algorithm. Due to the effect of instantaneous angular changes, it was seen that prediction errors with small magnitudes were occurring between the theoretical data and the QBP approach in the time intervals of the 4th and 6th seconds and the 10th and 12th seconds. When the prediction performance was evaluated in a general manner, it could be expressed that QBP could successfully predict the theoretical data for $\theta_{5}$.

When the proposed QBP-based approach was applied to the prediction of the sixth joint angle, it was seen that $\theta_{6}$ could successfully be predicted. As seen from Figure 23, QBP was able to produce a prediction performance that almost overlapped with the theoretical data during the entire time interval. The reason for this situation may be expressed as the lack of instantaneous angular changes or their occurrence with too small magnitudes.

**Figure 24:**
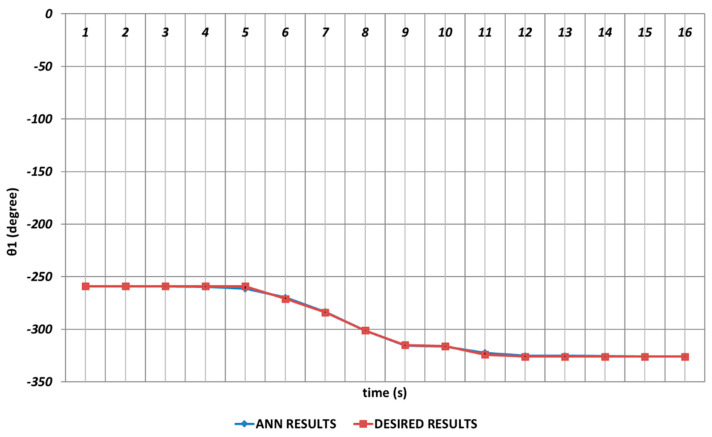
RBP learning algorithm-based prediction results for the first joint angle.

**Figure 25:**
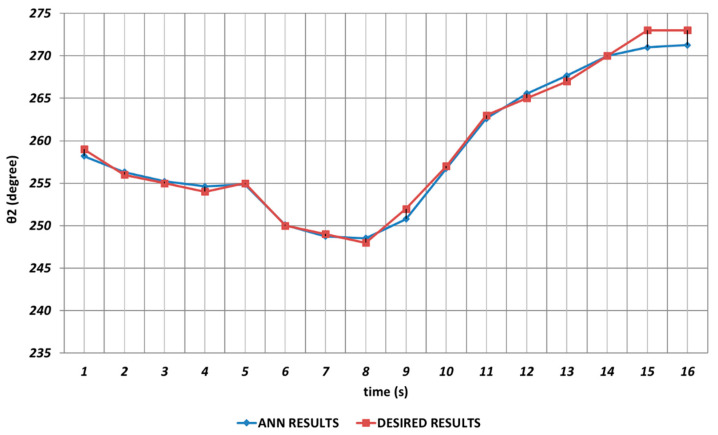
RBP learning algorithm-based prediction results for the second joint angle.

**Figure 26:**
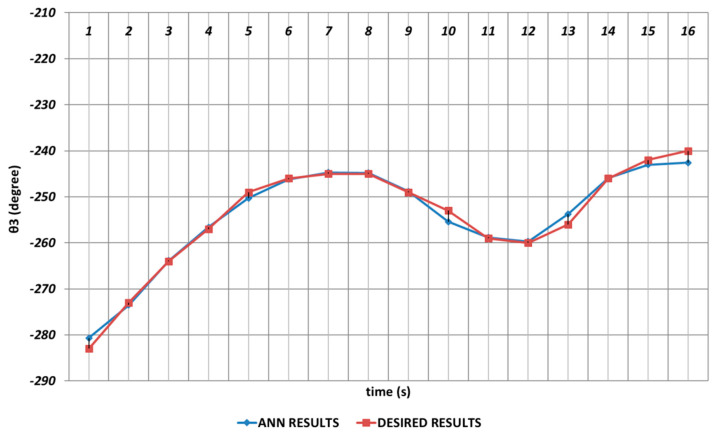
RBP learning algorithm-based prediction results for the third joint angle.

**Figure 27:**
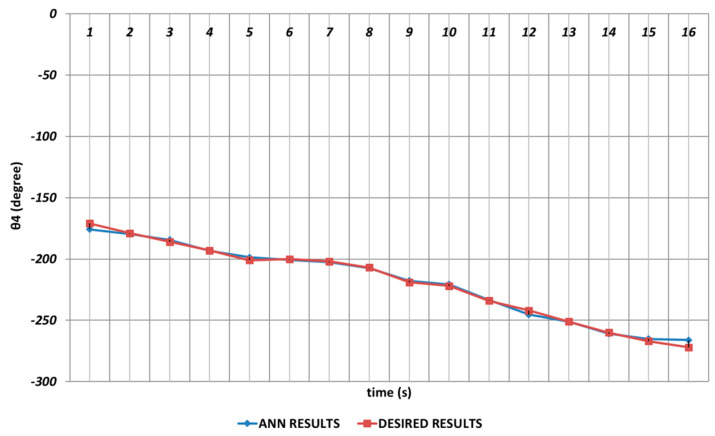
RBP learning algorithm-based prediction results for the fourth joint angle.

**Figure 28:**
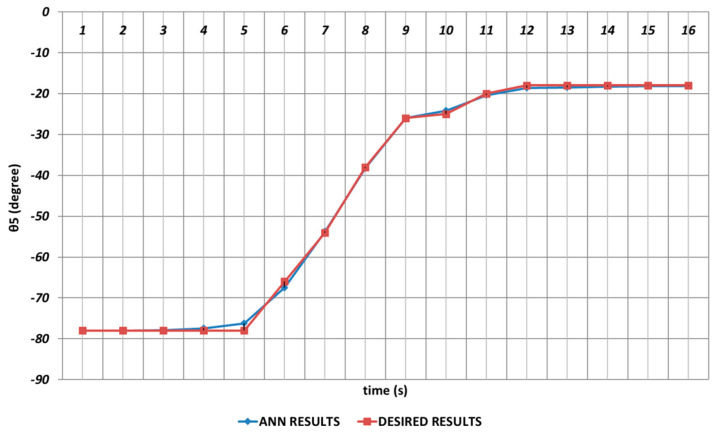
RBP learning algorithm-based prediction results for the fifth joint angle.

**Figure 29:**
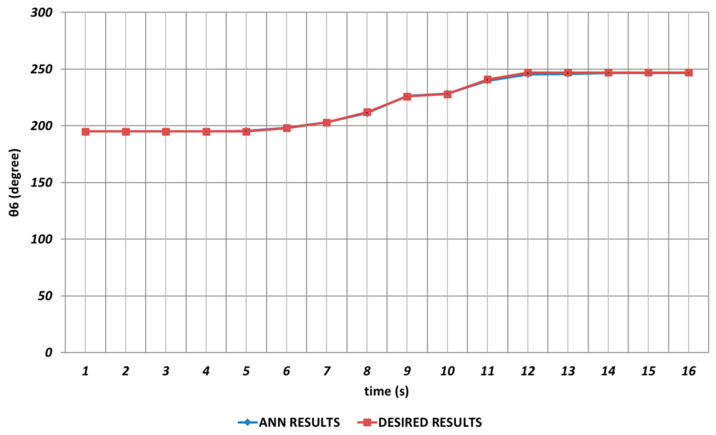
RBP learning algorithm-based prediction results for the sixth joint angle.

Figure 24, Figure 25, Figure 26, Figure 27, Figure 28 and Figure 29 represent the analysis results obtained by using the RBP learning algorithm. As seen from Figure 24, which shows the modeling performance for $\theta_{1}$, RBP was able to predict the theoretical data with high accuracy. Although there were instantaneous angular changes at the fourth and sixth seconds, the RBP could also converge to these changes successfully.

Figure 25 demonstrates the RBP-based prediction results for $\theta_{2}$. As seen from the figure, the deviations with high amplitudes from the theoretical data were occurring during the entire time interval. $\theta_{2}$ corresponded to the shoulder angle of the robot manipulator, and it was seen that the prediction performance of the RBP in terms of $\theta_{2}$ decreased significantly as a result of the instantaneous angular changes. In other words, it would be inevitable that the lack of high prediction performance for the second joint angle would cause significant errors in chemical applications in the production process.

In Figure 26, the prediction results obtained for $\theta_{3}$ by using the RBP-based ANN are shown. As in the prediction process of the second joint angle, the RBP produced worse prediction results during the entire time interval and also could not converge to the theoretical data of the $\theta_{3}$ joint angle enough. Namely, the basic RBP learning algorithm seemed insufficient in predicting the theoretical data of the third joint angle.

In predicting the $\theta_{4}$ joint angle, the RBP learning algorithm could not provide a completely adequate performance as shown in Figure 27. Especially, deviations having high magnitudes from the theoretical data were occurring in the time intervals of the 1st and 2nd seconds, the 11th and 13th seconds, and the 15th and 16th seconds due to the instantaneous angular changes. In addition, deviations having small magnitudes were also observed in the time intervals of the 4th and 7th seconds and the 8th and 11th seconds.

The RBP-based prediction performance for the $\theta_{5}$ joint angle is shown in Figure 28. Due to the effects of instantaneous angular changes, it was seen that prediction errors were occurring at unacceptable rates between the theoretical and RBP approaches in the time intervals of the 3rd and 7th seconds and the 9th and 16th seconds.

The prediction results obtained for $\theta_{6}$ are shown in Figure 29. From the figure, it can be seen that the RBP learning algorithm was able to provide an effective prediction performance during the entire time interval except the 11th and 14th seconds. It can also be concluded that the lack of instantaneous angular changes or their occurrence with too small magnitudes resulted in an effective prediction performance.

In order to present a more detailed analysis, in addition to the 3-10-6 ANN network structure including 10 nonlinear cells in the hidden layer, the performance of the 3-5-6 network structure, which included five nonlinear cells in its hidden layer, was also analyzed. The performances of the network structures were compared in terms of RMSE and statistical R^2^ metrics. The results obtained for RMSE are presented in Table 3. From the results, it was seen that that the network structure including five nonlinear cells in its hidden layer could provide a faster learning effect. However, it was also seen that as the number of nonlinear cells in the hidden layer decreased, the error performance and therefore the prediction performance decreased. When the results were evaluated in general, it could be expressed that the QBP produced the best prediction results for the 3-10-6 network structure, while the DBD produced the best prediction results for 3-5-6 network structure.

The statistical performances of the improved ANN network structures are another important performance metric. In this study, the R^2^ statistical analysis approach was applied to test the reliability and stability of the obtained results. In Equation (8) given for R^2^,

(8) $$R^{2}=1-\frac{\;\sum {\left(ideal_{i}-id\hat{e}al_{i}\right)}^{2}}{\sum {\left(ideal_{i}-id\tilde{e}al_{i}\right)}^{2}}$$

$ideal_{i}$ can be defined as the ideal values of the joint angles; $id\hat{e}al_{i}$ represents the joint angle values obtained from the regression equation, and finally, $id\tilde{e}al_{i}$ is the mean value of the ideal joint angles. The value of the R^2^ parameter changed in the interval of [0, 1]. The R^2^ values close to 1 proved the effectiveness of the model. From Table 4, which represents the statistical performances of each algorithm, it can be concluded that all the learning algorithms were able to provide statistically superior performance. In other words, each learning algorithm exhibited stable behavior by converging to approximately the same results at each run.

## 4. Conclusions

In this study, ANN-based positioning analyses were carried out to predict the joint angles of a 6-DOF industrial robot manipulator system for trajectory analysis. DBD, OBP, QBP, and RBP learning algorithm-based ANN network structures were improved and then applied for the prediction of six joint angles with high accuracy. The applicability of ANNs in multi-DOF robot manipulator trajectory prediction was demonstrated, and the superiority of ANN-based approaches in trajectory prediction was proved.

In addition to the 3-10-6 type ANN structure, the 3-5-6 type ANN structure was also improved and applied to the prediction trajectory analysis. The simulation results obtained for the 3-10-6 type ANN structure represented that all learning algorithms could successfully predict the $\theta_{1},\theta_{4},\theta_{5}$, and $\theta_{6}$ joint angles with high accuracy. When the prediction results obtained for these joint angles were examined, it was seen that instantaneous deviations were occurring only at certain time intervals and with low magnitudes. On the other hand, the prediction performances of the algorithms for $\theta_{2}$ and $\theta_{3}$ seemed too weak due to the instantaneous deviations with high magnitudes that occurred during the entire time interval. 

In order to present a detailed performance comparison between the 3-10-6 and 3-5-6 type ANN structures, the RMSE and the reached statistical R^2^ values were also compared. The mean and maximum RMSE values reached by the algorithms represented that QBP produced the best results for the 3-10-6 ANN structure, and DBD produced the best results for the 3-5-6 ANN structure. When the results were evaluated in terms of the RMSE performance, it could be expressed that RBP produced the worst results among the four learning algorithms. The R^2^ values reached by the algorithms showed that each learning algorithm was able to produce similar statistical performances, but the reliability and stability of the DBD-based ANN structure seemed a bit better when compared with other learning algorithms.

Consequently, it can be stated that the ANN-based approaches proposed in this study can be used effectively even in the optimal trajectory analysis of real-time robot manipulators operating under large disturbances.

In future studies, deep learning-based controllers will be improved to optimize the trajectory of multi-DOF robot manipulators. Especially, it will be aimed to construct deep learning-based models for the robot manipulators having more than 6 DOF.

## Figures and Tables

**Table 1:** The technical properties of the *Universal Robots UR5e* model 6-DOF robot manipulator.

Weight	18.4 kg
Payload capacity	5 kg
Reachability distance	850 mm
Max-Min rotation angles	+/− 360° at all joints
Angular velocity	Joints—maximum 180° per second Vehicle—about 1 m per second
Repeatability	+/− 0.1 mm
Volume diameter	Ø 149 mm
Number of joints	6 rotational joints
Control box size	475 mm × 423 mm × 268 mm (W × H × D)
I/O ports	18 digital inputs, 18 digital outputs, 4 analog inputs, 2 analog outputs
I/O power supply	24V-2A in control box and 12V/24V-600 mA in vehicle
Communication	TCP/IP—Modbus TCP
Programming	Polyscope graphical user interface on 12-inch touch screen
Noise	Low noise levels
IP classification	IP54
Power consumption	≅200 W
Materials	Aluminum, PP plastic
Working temperatures	0–50 °C
Power source	100–240 V (AC), 50–60 Hz

**Table 2:** D-H parameters of the Universal Robots UR5e model 6-DOF robot manipulator.

Number of Joint	Two Link Twist Axes Angle	Link Length	Link Offset	Joint Angle	Joint Variable
*i*	*α_i_*_−1_	*a_i_*_−1_	*d_i_*	*Θ_i_*	*d_i_* or Θ_i_
1	0	L_1_	0	Θ_1_	Θ_1_
2	0	L_2_	0	Θ_2_	Θ_2_
3	0	L_3_	0	Θ_3_	Θ_3_
4	0	L_4_	0	Θ_4_	Θ_4_
5	0	L_5_	0	Θ_5_	Θ_5_
6	0	L_6_	0	Θ_6_	Θ_6_

**Table 3:** Performance comparison of the used ANN network structures.

Learning Algorithm	ANN Network Structure	Mean RMSE (Training)	Maximum RMSE (Training)	Mean RMSE (Test)	Maximum RMSE (Test)
DBD	3-10-6	0.427328	1.20820	0.418576	1.20820
OBP	3-10-6	0.485862	1.20600	0.507729	1.20600
QBP	3-10-6	0.373176	1.19047	0.374058	1.19047
RBP	3-10-6	1.210040	2.86877	1.267080	1.27537
DBD	3-5-6	0.726305	1.60402	0.734876	1.34779
OBP	3-5-6	0.826176	1.73226	0.804328	1.73226
QBP	3-5-6	0.878670	1.98618	0.911634	1.98618
RBP	3-5-6	1.385830	2.72113	1.523790	2.72113

**Table 4:** R^2^ values obtained for each learning algorithm.

Learning Algorithm	ANN Network Structure	$R^{2}$					
		$\theta_{1}$	$\theta_{2}$	$\theta_{3}$	$\theta_{4}$	$\theta_{5}$	$\theta_{6}$
DBD	3-10-6	0.99997	0.99938	0.99773	0.99932	0.99996	0.99996
OBP	3-10-6	0.99996	0.99870	0.99310	0.99953	0.99995	0.99990
QBP	3-10-6	0.99900	0.99870	0.99310	0.99953	0.99995	0.99990
RBP	3-10-6	0.99900	0.98952	0.98709	0.99426	0.99934	0.99910
DBD	3-5-6	0.99957	0.99734	0.99349	0.99853	0.99946	0.99985
OBP	3-5-6	0.99950	0.99803	0.99355	0.99824	0.99933	0.99933
QBP	3-5-6	0.99993	0.99602	0.99449	0.99595	0.99994	0.99995
RBP	3-5-6	0.99922	0.97655	0.96693	0.99360	0.99915	0.99854

## Data Availability

Data are contained within the article.
